# Using the Framework for Reporting Adaptation and Modifications-Expanded (FRAME) to Adapt an Evidence-Based HIV-Prevention Intervention for Zambian Adolescent Girls and Young Women

**DOI:** 10.1007/s10461-026-05033-5

**Published:** 2026-01-27

**Authors:** Gina Diagou Sissoko, Tukiya Kanguya, Mwamba Mwenge, Anjali Sharma, Nok Chhun, Stephanie H. Yu, Erin Emerson, Sybil Hosek, Carolyn Bolton-Moore, Geri R. Donenberg

**Affiliations:** 1 Center for Dissemination and Implementation Science, Department of Medicine, University of Illinois at Chicago, Chicago, IL USA; 2 Centre for Infectious Disease Research in Zambia, Lusaka, Zambia; 3 Department of Psychiatry, University of California San Francisco, San Francisco, CA USA; 4 University of Alabama at Birmingham, Birmingham, AL USA; 5 Center for Interdisciplinary Research on AIDS (CIRA), School of Public Health, Yale University, 60 College St, New Haven, CT 06510 USA

**Keywords:** HIV prevention, Adolescent girls and young women, Zambia, FRAME, Intervention adaptation, Sexual health, Implementation science

## Abstract

Despite substantial global efforts, adolescent girls and young women (AGYW) in Southern Africa remain disproportionately affected by HIV. In Zambia, the HIV prevalence among AGYW is nearly three times that of their male peers. The Informed Motivated Aware and Responsible Adolescents and Adults (IMARA) program, an evidence-based HIV/STI prevention intervention initially developed in the United States and later adapted in South Africa, demonstrated promising reduction in HIV risk through culturally tailored, mother-daughter–focused strategies. This paper outlines the process of adapting IMARA for Zambian AGYW by utilizing the Framework for Reporting Adaptations and Modifications-Expanded (FRAME). The adaptation process consisted of four phases: (1) Assessment through stakeholder meetings and focus groups involving adolescents and caregivers; (2) Intervention Delivery Decisions guided by community feedback; (3) Curriculum Adaptation through iterative revisions and collaborative input from Zambian and U.S.-based researchers; and (4) Theater Testing with adolescent–caregiver dyads to refine the content. All modifications were documented using FRAME. Eight key modifications, covering content, context, and training, were implemented to create ZAIMARA (Zambian Informed Motivated Aware and Responsible Adolescents and Adults). These changes were proactively planned, occurred pre-implementation, and were deemed fidelity-consistent. Examples include translating materials into a local language and changing intervention terms and scenarios to reflect cultural norms. Applying FRAME to adapt IMARA in Zambia provided a transparent, systematic method to maintain cultural relevance with fidelity to an evidence-based intervention. Our study demonstrates the utilization of the FRAME for future adaptations of HIV prevention programs in diverse global contexts.

## Introduction

Despite significant global efforts to reduce the spread of HIV/AIDS, adolescent girls and young women (AGYW) in Southern Africa continue to bear a disproportionate burden of new infections, which is estimated at 77% of all new HIV infections [1]. In Zambia, HIV prevalence among 15–19-year-old AGYW is approximately three times higher than their male peers at 5.7% compared to 1.8% [2]. However, 70.1% of AGYW living with HIV are unaware of their status, and only 45.4% have ever been tested for HIV [2].

Several factors contribute to the heightened vulnerability of Zambian AGYW to HIV. Although 63% of AGYW are sexually active, only 34% reported using condoms during their last sexual encounter [2]. Moreover, 3.6% experienced vaginal discharge, and 2.8% had a vaginal ulcer [2], which may indicate a sexually transmitted infection (STI). Zambia’s syndromic treatment approach (versus confirmed diagnoses) to STIs often leaves asymptomatic infections undetected and untreated, exacerbating health risks for AGYW [2]. Despite the provision of free prevention services, including pre-exposure prophylaxis (PrEP), uptake is suboptimal among AGYW in Zambia [3].

The Zambian government noted the urgency of addressing HIV prevention and sexual health among AGYW and prioritized them in its national health strategy [4–6]. Effective efforts to address HIV, however, require multilevel, comprehensive prevention packages that are integrated, synergistic, and tailored to the local epidemiological and cultural context [7–12]. Addressing persistent HIV disparities among AGYW necessitates interventions that include a nuanced focus on the social and structural inequities that drive risk behaviors and continue to exacerbate new infection rates [5, 6].

### IMARA as a Promising Intervention

IMARA (Informed Motivated Aware and Responsible Adolescents and Adults) is a CDC-recognized "best-evidence" intervention that aims to reduce HIV and STI incidence, increase HIV testing, and promote pre-exposure prophylaxis (PrEP) uptake among AGYW. IMARA leverages the mother-daughter relationship to prevent HIV acquisition by empowering mother figures—defined as female caregivers, such as biological mothers, grandmothers, or aunts—as trusted guides to promote safer sexual behaviors and greater uptake of HIV prevention services [8].

IMARA employs a multifaceted approach to strengthen the mother-daughter bond, promote the adoption of prevention behaviors, and support community-level shifts towards safer norms and reduced stigma. The intervention has been previously detailed [8, 12]. IMARA is delivered over two days in a group and integrates culturally relevant and gender-specific approaches to address the unique challenges faced by AGYW and their female caregivers (FC). Session components are conducted both separately and jointly, with parallel content in different groups focused on individual, sociocultural, and structural factors that contribute to HIV and STI risk. Session components empower participants to explore topics like self-efficacy, relationship dynamics, and risk reduction strategies. Joint mother-daughter activities build on these foundations to foster open, effective communication, enhance trust, and practice new skills together. IMARA incorporates the influence of mental health challenges and substance use on sexual behaviors that increase vulnerability to HIV, with the aim of providing participants with the tools to mitigate these risks.

IMARA’s effectiveness was first demonstrated in a U.S.-based group randomized controlled trial involving 14–18-year-old Black AGYW. The intervention showed a 43% reduction in incident STIs at 12-month follow-up compared to a control group [8]. IMARA was subsequently adapted for 15–19-year-old AGYW in South Africa (IMARA-SA) [12, 13], where it demonstrated strong implementation and positive preliminary outcomes (e.g., increase PrEP uptake, reductions in sexual risk behaviors, favorable trends in STI incidence and mental health outcomes) [12, 14]. This prior adaptation highlighted the importance of culturally tailoring interventions and positioned IMARA-SA as a promising model for HIV prevention among AGYW in Zambia.

### Applying the Framework for Reporting Adaptations and Modifications (FRAME)

Adapting evidence-based interventions to new settings is critical to maintain their efficacy and ensure cultural and contextual relevance [15, 16]. This process requires balancing fidelity to the core components of the intervention (e.g., increasing sex-related communication between AGYW and female caregivers) with flexibility to address the unique sociocultural, epidemiological, and structural factors of the target population [17]. The Framework for Reporting Adaptations and Modifications-Expanded (FRAME) offers a structured approach to document and guide the adaptation of evidence-based interventions to new settings [18, 19]. Careful documentation of adaptations is needed to strengthen implementation science research and help guide future adaptation efforts [20]. The use of FRAME is particularly significant as it addresses the historical lack of standardized methods to document intervention adaptations [19], thereby offering clarity and consistency that enhance both replicability and sustainability [17]. FRAME comprises eight core domains that provide a comprehensive structure for documenting adaptations (see Table 1).

**Table 1:** FRAME core components

Modification domain	Descriptions
What is modified	Specifies the areas of modification, including content (e.g., intervention materials or activities), context (e.g., delivery setting), or implementation processes (e.g., training or evaluation)
Who participates in modifications	Captures the individuals or groups involved in recommending or deciding on adaptations. These may include practitioners, administrators, policymakers, researchers, or stakeholders
When the modification occurred	Describes timeline of modifications, which can occur at different stages of implementation, including pre-implementation (e.g., pilot testing), active implementation, and scale-up or sustainment phases
Planned or unplanned nature of modifications	Distinguishes between proactive (planned) adaptations, made to anticipate challenges or improve fit, and reactive (unplanned) modifications, implemented in response to unforeseen circumstances
Fidelity considerations	Discusses whether modifications align with the intervention’s core components (fidelity-consistent) or deviate from them in a way that could compromise effectiveness (fidelity-inconsistent). An "unknown" category accounts for cases where the impact on fidelity is unclear
Level of delivery	Identifies the scope of modifications, such as changes targeting individual recipients, specific subgroups, entire organizations, or broader systems
Extent of modification	Discusses extent of modification, as adaptations may range from transient, context-specific changes to permanent, widespread alterations
Why the modification was made (rationale)	Documents the reasons for modifications and rationale categories namely improving fit, increasing engagement, addressing cultural relevance, managing constraints, or enhancing feasibility

*FRAME* Framework for Reporting Adaptations and Modifications-Expanded

### Current Paper

This paper documents the adaptation of IMARA-SA for Zambian AGYW using FRAME to ensure a systematic and rigorous process. Utilizing the FRAME to document IMARA-SA’s adaptation for Zambian AGYW contributes to a growing literature on intervention adaptations in general and IMARA specifically (see Crooks et al. [21] and Merrill et al. [22] for adaptations of IMARA for Black male caregivers and Latinas, respectively). By showcasing the application of the FRAME to the adaptation process for IMARA within the Zambian context, we aim to highlight its utility as a model for documenting future adaptations, which are critical for achieving sustainable, long-term impact in global HIV prevention efforts.

## Methods

IMARA-SA was adapted for the Zambian context as part of a hybrid effectiveness implementation trial (Donenberg et al., 2025). All modifications reported in this paper occurred pre-implementation (i.e., before curriculum finalization and study recruitment) and involved collaborations across a global research team based at the Centre for Infectious Disease Research in Zambia (CIDRZ), U.S.-based researchers from the University of Illinois Chicago, and Zambian community members. A core adaptation team included the two principal investigators, co-investigators, project coordinators, and Zambian and U.S. research staff. The team coded and tracked all modifications during the adaptation process according to the FRAME domains. Modifications were discussed among the adaptation team members to ensure fidelity and reach consensus. While FRAME guided documentation of the changes, we followed the eight steps of the ADAPT-ITT framework [9] to identify needed adaptations. ADAPT-ITT is a systematic process used to tailor evidence-based interventions for cultural relevance and effective implementation in new contexts. Stakeholder engagement occurred throughout the pre-implementation period and included multiple mechanisms: recurring, separate consultations with CIDRZ’s adolescent and adult community advisory boards (CABs), discussions with the Zambian Ministry of Health Adolescent Technical Working Group, meetings with representatives from the Ministry of Health, and exploratory focus groups with AGYW and female caregivers at potential study sites. This multi-level engagement created methodological triangulation across youth, caregiver, community, and health system perspectives, which supported data adequacy for determining the necessary cultural and contextual adaptations.

The pre-implementation adaptation process occurred from October 2022 to February 2025. The initial pre-award period focused on determining fit and feasibility for the Zambian context, which informed the decision to pursue funding from the National Institutes of Health (NIH). After meetings with various stakeholder groups outlined above, and receiving overwhelmingly positive feedback, we applied for and received funding from the National Institute of Child Health and Human Development in September 2023. The formal adaptation process began in October 2023.

Below, we document the modifications that occurred during five ADAPT-ITT steps: (1) Assessment, (2) Intervention Delivery Decisions, (3) Curriculum Adaptation, and (4) Theater Testing, culminating in a final version of the curriculum used for the (5) Pilot Test.

### Step 1: Assessment

The Assessment step occurred in two phases, one before and one after research funding was awarded. The process included multiple stakeholder engagement mechanisms, including meetings with key informants and focus groups with a small grant from the Fogarty International Center. The U.S.-based investigator traveled to Zambia to meet with CIDRZ investigators to determine whether IMARA was appropriate for the Zambian context. During the visit, the team held several stakeholder meetings with key informants, including separate meetings with CIDRZ’s adolescent and adult clinical research CABs, and a meeting with the Zambian Ministry of Health Adolescent Technical Working Group. The investigators also met with representatives from the national Ministry of Health as part of this early consultative process. Additionally, we conducted two focus groups of 8–10 AGYW and female caregivers, respectively, at two potential research sites, where we described IMARA activities. These focus groups were designed to complement, rather than constitute, the primary source of adaptation input.

Individuals shared perceptions of community needs, cultural factors relevant to HIV prevention with AGYW, and the feasibility, appropriateness, and acceptability of delivering IMARA in Zambia. This multi-source engagement generated a broad understanding of contextual considerations and informed the decision to pursue a full trial. Based on positive feedback, the investigators applied for funding from the National Institutes of Health to support a large research study testing IMARA’s effectiveness and implementation. Funding was awarded a year later.

The second phase of the Assessment occurred after funding was received. During the post-award period, the research team re-convened the stakeholder groups to obtain more specific feedback on the curriculum components, and this time, employed rigorous methods and kept detailed documentation. Specifically, adult community and youth advisory board members were separately presented with the full IMARA curriculum to provide feedback on each described activity. The second phase of the Assessment revealed clear guidelines for necessary adaptations for language, cultural beliefs, logistical structures, and contextual relevance. These post-award consultations offered deeper, activity-by-activity input and directly informed Step 2 of the ADAPT-ITT process.

### Step 2: Intervention Delivery Decisions

Following the first Assessment phase, the research team—comprised of principal investigators, co-investigators, research staff, and study coordinators—discussed recommendations from the Assessment. We decided *how* IMARA would be delivered in Zambia (e.g., locations, facilitators, duration of sessions) and initial modifications to the curriculum to accommodate Zambian cultural norms. Investigators determined the training needs for peer facilitators and the broader implementation strategy. Final decisions on the overall scope (delivery, training, and population focus) and process were substantially informed by community member feedback obtained during the Assessment step.

### Step 3: Curriculum Adaptation

In step 3, we began curriculum modifications. We met with CIDRZ’s adult and youth CABs as needed. We presented the IMARA-SA curriculum and elicited reactions and feedback. Over eight months, the study coordinator and one co-investigator met weekly, with periodic inputs from the PI, to review the proposed modifications. All changes were systematically documented and coded using the FRAME. Drafts of the adapted curriculum were continuously reviewed by the principal investigators to ensure fidelity to IMARA-SA’s core components. In parallel, youth and adult CABs convened twice to review and provide feedback on the revised materials. This iterative process facilitated consensus on curriculum content, delivery methods, and session activities prior to theater testing. By the end of step 3, a near-final version of the adapted curriculum was prepared for theater testing.

### Step 4: Theater Testing

At this step, we conducted theater testing to evaluate modifications and refine the curriculum. We pre-selected modules with potential cultural concerns to theater test (e.g., assertive communication, sex education, including on auto-erotic/non-penetrative and penetrative sex). We recruited eight adolescent–caregiver dyads from communities with similar sociocultural and demographic characteristics as our study sites. In a 1-day session, the study team presented pre-selected modules (six for AGYW, five for FCs, seven jointly) to AGYW and female caregivers. Immediately following each presentation, AGYW and female caregivers provided structured (via evaluation forms) and open-ended feedback on clarity, cultural appropriateness, acceptability, feasibility, and overall satisfaction. Research assistants documented feedback provided during the focus group discussion at the end of the session. Focus groups were conducted separately for the AGYW and female caregivers to promote honest communication and avoid social desirability bias. Following the theater testing, Zambian-based researchers reviewed participant feedback, discussed with the full research team, and made final minor changes to the curriculum in preparation for the Pilot Study.

## Results

Eight modifications were made to IMARA-SA to form ZAIMARA (See Table 2). All modifications were proactive, planned, and occurred before implementation [19]. Changes were determined to be fidelity-consistent because the core component aims were not changed (e.g., increasing communication and sex education). Consistent with the FRAME structure, all adaptations were thematically categorized as either content modification (*n* = 6) or contextual modifications (*n* = 2). Below, we describe the adaptations based on this categorization.

**Table 2:** Modifications for ZAIMARA using FRAME

	Modification	What	Who	Delivery level	Why/goal
1	Language change to Bemba	Content	RT	Entire target group	Fit
2	Curriculum Motto was changed	Content	RT, CM	Entire target group	Fit Cultural Factors
3	IMARA poem was changed	Content	RT	Entire target group	Fit Cultural Factors
4	IMARA theme song was changed	Content	CM RT	Entire target group	Fit Cultural Factors
5	Intervention terms were changed (i.e., assertive to effective communication)	Content	RT, CM	Entire target group	Cultural Factors Satisfaction Retention Effectiveness/outcomes
6	Names and scenarios changed to Zambian context	Content	RT, CM	Entire target group	Fit Cultural Factors
7	Increase session length on communication to 150 min	Context	RT, CM	Entire target group	Reach/engagement Effectiveness/outcomes
8	Mother figure recipients were expanded to include younger female caregivers	Context	RT, CM	Entire target group	Reach/engagement

All reported modifications were planned, occurred pre-implementation (before study recruitment), are considered fidelity-consistent; *RT* research team, *CM* community members (including community advisory board members and theater testing participants)

### Content Modifications

FRAME conceptualizes content modifications as “changes made to the intervention procedures, materials, or delivery” [18]. Six content-related adaptations were introduced in ZAIMARA. First, the intervention curriculum language was expanded from the national language English to include iciBemba and iciNyanja, which study investigators viewed as essential to improving fit for Zambian adolescents and female caregivers. Several adolescents and caregivers emphasized that they express themselves more naturally and comfortably in iciBemba or iciNyanja, particularly when discussing family relationships or sexual health topics.

Second, the research team and youth CAB expanded the IMARA motto to reflect the culture-specific adaptation made for Zambia (ZA) to now read as “ZAIMARA is mothers and daughters. ZAIMARA is staying safe. ZAIMARA is healthy living. We are ZAIMARA!” The adolescent CAB, in particular, felt that this change would increase Zambian identification with the program. Third, IMARA-SA included a South African theme song related to empowerment. To increase engagement and cultural fit, based on the suggestion of the adolescent CAB, the song was changed to a popular Zambian song celebrating female empowerment in the context of HIV and STI prevention. This song was selected by CIDRZ adolescent CAB. Fourth, IMARA-SA contained an empowerment poem by a prominent African American author and poet. In collaboration with the adolescent and adult CAB, we substituted a locally authored poem to better reflect the current challenges AGYW and female caregivers face in Zambia.

Fifth, certain caregiver–AGYW communication scenarios were amended to reflect Zambian names and culturally acceptable dialogue. Initial drafts contained language that some adolescents felt they would never use with their caregivers, and some caregivers—particularly fathers who attended CAB discussions—believed the scenarios depicted disrespectful speech. Specifically, adolescents explained that some of the original IMARA-SA conversation scripts reflected interaction styles that did not resemble how they communicate at home. They described certain scenarios and phrases as too direct for Zambian norms, especially in conversations involving dating and sexual health. Caregivers similarly indicated that some dialogue felt disrespectful or misaligned with expected hierarchies within families, reinforcing the need to revise tones and phrasing. To resolve these concerns, the research team convened a separate session with female caregivers and AGYW, who reviewed and revised content related to romantic or sexual conversations they considered unsuitable for caregiver–AGYW exchanges.

Sixth, the term “assertive communication” was replaced with “effective communication,” because “assertive” was viewed as conflicting with local norms governing respectful discourse between youth and adults. During the CAB consultations, caregivers and youth emphasized that “assertiveness” as defined in Western communication models risked being misinterpreted as confrontational in the Zambian context. They expressed that an emphasis on calm delivery and respect better captures how adolescents are expected to communicate with adults. As such, renaming the skill “effective communication” (which is a core component of the IMARA intervention) aligned the curriculum with the cultural expectations while preserving the intervention’s behavioral intent. These content adaptations sought to address cultural fit, norms about family communication, participant acceptability and satisfaction, and anticipated retention.

### Context Modifications

Context modifications involve the setting or location in which the intervention is delivered, the format or channel of delivery, the personnel who deliver the intervention, or the population receiving it [18]. In ZAIMARA, researchers made two modifications related to context. First, the intervention broadened its definition of female caregiver to allow younger caregivers (e.g., cousins and sisters) provided they met inclusion criteria based on community members’ observation that many adolescent girls seek guidance from older relatives who are closer in age. This change was intended to expand overall reach and increase engagement in the project.

Second, the length of the effective communication module was increased from 120 to 150 min based on participant feedback during theater testing asking for more time to discuss and understand the communication concepts. Citing discomfort and lack of skills navigating discussions on sexual education, caregivers suggested that providing female caregivers with more guidance on how to engage in open, non-judgmental conversations would be beneficial. Thus, increased time on the communication module was anticipated to foster more productive dialogues and help female caregivers overcome cultural and emotional barriers preventing them from addressing these critical issues with their daughters. The change is intended to increase the intervention’s engagement and effectiveness.

## Discussion

In this paper, we describe the application of a rigorous documentation approach, FRAME, to the systematic adaptation of IMARA-SA for Zambian AGYW and their female caregivers. The modifications illustrate how content and context adaptations were made in a family-based HIV prevention program implemented in a resource-constrained setting. All adaptations to existing interventions must balance cultural and contextual relevance with fidelity to core intervention components [9, 15, 17]. We did this by adapting IMARA-SA in a way that challenges existing cultural norms (e.g., lack of direct communication between AGYW and female caregivers) in an acceptable manner. Although the modifications presented here were primarily face-valid rather than substantive, each was designed to strengthen the acceptability, feasibility, and appropriateness of the intervention for Zambian AGYW and their female caregivers, for whom structural barriers and social norms often limit engagement in HIV prevention efforts.

All modifications were enacted before the study began (i.e., pre-implementation), and they were proactively planned, guided by input from diverse community partners and AGYW themselves. All modifications were assessed by the team and were deemed fidelity-consistent. This is noteworthy, as prior research emphasizes that poorly documented or reactive adaptations can undermine the effectiveness of evidence-based interventions [19]. In this adaptation, fidelity-consistency was determined by systematically evaluating each proposed modification against IMARA’s core components and underlying mechanisms of action (e.g., open communication). The adaptation team reviewed whether changes altered the intervention’s targets, behavioral processes, or structural elements—and only modifications that preserved the core features were implemented. This approach allowed us to preserve IMARA’s core emphasis on a female caregiver who can facilitate and support safer sexual behaviors—while addressing cultural considerations in Zambia. For example, changing key terms (e.g., from “assertive” to “effective” communication) and adjusting communication scenarios represent important efforts to align ZAIMARA with Zambian sociocultural norms surrounding family hierarchies and respectful discourse. Both have been identified as significant determinants of intervention uptake [23]. Our analytic adaptation process ensured enhanced cultural relevance and acceptability without compromising the integrity of the original evidence-based model.

This paper expands previous research on intervention adaptations by applying a comprehensive framework to document the adaptation process [19]. There is growing recognition that the science of adaptation must be strengthened to ensure careful attention to core concepts and to guide future modifications. Our findings underscore that continuous community engagement is indispensable for identifying how best to integrate local values, which may include a greater local tolerance for cultural disruption than is typically assumed by social and behavioral scientists, whether considering emic or etic perspectives.

Importantly, the process of adaptation often brings into question the balance between internal and external validity. Internal validity—ensuring that observed effects can be attributed to the intervention’s core components—is traditionally prioritized in efficacy studies. However, when interventions are adapted for new cultural contexts, enhancing external validity (i.e., the generalizability and real-world applicability of the intervention) becomes crucial. In our adaptation of IMARA for Zambian AGYW, we were mindful of this balance. By employing the FRAME model, we systematically documented fidelity-consistent modifications, ensuring that the core elements that drive intervention efficacy were maintained even as the curriculum was tailored to better align with local cultural norms and community practices.

Thus, rather than sacrificing internal validity for external relevance, our approach illustrates that a rigorously documented adaptation process can successfully enhance cultural fit and community engagement without compromising the intervention’s integrity. This process demonstrates that local communities may indeed be more accepting of culturally disruptive elements—when these elements are thoughtfully adapted and supported by community input—than external experts might assume. This insight is essential not only for improving the immediate intervention outcomes but also for guiding future adaptations across diverse settings. By systematically documenting each modification using FRAME, we also contribute to the scant but increasing literature on standardized reporting of adaptations, which is key to comparing and replicating interventions across diverse settings [17, 19, 24].

### Implications for Research and Practice

Documenting the adaptation process offers several implications for researchers and practitioners. First, explicit documentation enhances transparency by detailing the rationale behind modifications and enabling a nuanced understanding of how these cultural adaptations affect participant engagement and intervention fidelity. This rigor not only facilitates replication and comparison across settings but also aids in evaluating which specific changes contribute most to improved outcomes. Furthermore, while the adaptations reported here occurred before the randomized trial began, ongoing documentation as real-world challenges emerge during implementation is critical. Systematically tracking subsequent modifications—along with analyzing their impact on both fidelity and outcomes—will be essential for guiding large-scale implementation by ensuring that adaptations remain effective and culturally relevant over time.

### Limitations

Study limitations warrant attention. First, modifications occurred during the pre-implementation phase and do not account for peri- and post-implementation modifications that will almost certainly occur during pilot testing and study rollout. We will document additional changes over time. Second, although we categorized our modifications as fidelity-consistent, future empirical evaluation is needed to confirm that these changes do not diminish intervention effectiveness. Third, it is unclear whether documenting the adaptations described here will impact long-term adaptation or sustainability metrics; ongoing evaluations will help clarify how adaptation documentation will benefit the field of intervention adaptation over time.

### Future Directions

As we pursue pilot testing to a fully powered hybrid effectiveness-implementation trial, future research should not only assess ZAIMARA’s impact on key HIV prevention outcomes—such as condom use, PrEP uptake, and STI testing rates—but also further refine our understanding of how to document and respond to adaptations that occur during trial rollout. In particular, systematically tracking mid-implementation and unplanned modifications using FRAME and similar approaches will be essential. Such documentation can offer critical insights into balancing intervention fidelity with enhancing cultural fit, thereby guiding real-time adjustments and informing best practices for future interventions.

Moreover, evaluating cost-effectiveness, fidelity maintenance, and the scalability of the adapted intervention will be crucial for health policymakers aiming to integrate gender-sensitive HIV prevention strategies at a national level [5]. By incorporating rigorous documentation methods throughout the intervention’s lifecycle—from initial adaptation to full-scale implementation—future studies can contribute to a growing body of evidence on the practical and theoretical benefits of systematic adaptation tracking, ultimately strengthening the science of intervention adaptation in dynamic, real-world contexts.

## Conclusions

Applying FRAME to systematically document adaptations to IMARA for Zambian AGYW enhances transparency and contributes to the science of cultural adaptation in HIV research. This report adds to a growing literature on the importance of documenting culturally and contextually anchored HIV prevention interventions in new settings while retaining fidelity to core components. As the global HIV epidemic continues to affect young women and girls disproportionately, FRAME provides a robust approach to documentation of equitable, sustainable, and effective intervention adaptations.

## Data Availability

The documentation and materials generated during the current study are not publicly available but are available from the senior author upon reasonable request.

## Abbreviations

ADAPT-ITT: Assessment, Decision, Adaptation, Production, Topical Experts, Integration, Training, Testing (Framework for adapting evidence-based interventions)
AGYW: Adolescent Girls and Young Women
CAB: Community Advisory Board
CM: Community Members
CIDRZ: Centre for Infectious Disease Research in Zambia
FC: Female Caregivers
FRAME: Framework for Reporting Adaptations and Modifications-Expanded
HIV: Human Immunodeficiency Virus
IMARA: Informed, Motivated, Aware and Responsible Adolescents and Adults
IMARA-SA: IMARA South Africa
PrEP: Pre-Exposure Prophylaxis
RT: Research Team
STI: Sexually Transmitted Infection
UNAIDS: Joint United Nations Programme on HIV/AIDS
ZAIMARA: Zambian Informed, Motivated, Aware and Responsible Adolescents and Adults
